# Deletional Alpha-Thalassemia Alleles in Amazon Blood Donors

**DOI:** 10.1155/2020/4170259

**Published:** 2020-04-14

**Authors:** Fernanda Cozendey Anselmo, Natália Santos Ferreira, Adolfo José da Mota, Marilda de Souza Gonçalves, Sérgio Roberto Lopes Albuquerque, Nelson Abrahim Fraiji, Ana Carla Dantas Ferreira, José Pereira de Moura Neto

**Affiliations:** ^1^Fundação Hospitalar de Hematologia e Hemoterapia do Amazonas, Manaus, Amazonas, Brazil; ^2^Universidade Federal do Amazonas, Faculdade de Ciências Farmacêuticas, Manaus, Amazonas, Brazil; ^3^Universidade Federal do Amazonas, Faculdade de Ciências Agrárias, Manaus, Amazonas, Brazil; ^4^Fundação Oswaldo Cruz–Centro de Pesquisas Gonçalo Moniz, Salvador, Bahia, Brazil

## Abstract

Alpha-thalassemia is highly prevalent in the plural society of Brazil and is a public health problem. There is limited knowledge on its accurate frequency and distribution in the Amazon region. Knowing the frequency of thalassemia and the prevalence of responsible mutations is, therefore, an important step in the understanding and control program. Hematological and molecular data, in addition to serum iron and serum ferritin, from 989 unrelated first-time blood donors from Amazonas Hemotherapy and Hematology Foundation (FHEMOAM) were collected. In this study, the subjects were screened for −*α*^3.7/4.2^/^20.5^, −^SEA,^ −^FIL^, and −^MED^ deletions. Alpha-thalassemia screening was carried out between 2016 and 2017 among 714 (72.1%) male and 275 (27.9%) female donors. The aims of this analysis were to describe the distribution of various alpha-thalassemia alleles by gender, along with their genotypic interactions, and to illustrate the hematological changes associated with each phenotype. Amongst the patients, 5.35% (*n* = 53) were diagnosed with deletion –*α*^−3.7^ and only one donor with *α*^−4.2^ deletion. From the individuals with –*α*^−3.7^, 85.8% (*n* = 46) were heterozygous and 14.20% (*n* = 7) were homozygous. The frequency of the –*α*^−3.7^ deletion was higher in male (5.89%) than in female (4.0%). There is no significant difference in the distribution of –*α*^−3.7^ by gender (*p* = 0.217). The –*α*^20.5^, −^SEA^, and −^MED^ deletions were not found. All subjects were analyzed for serum iron and serum ferritin, with 1.04% being iron deficient (*n* = 5) and none with very high levels of stored iron (>220 *µ*g/dL). Alpha-thalassemia-2^3.7kb^ deletion was the most common allele detected in Manaus blood donors, which is a consistent result, once it is the most common type of *α*-thalassemia found throughout the world. As expected, the mean of hematological data was significantly lower in alpha-thalassemia carriers (*p* < 0.001), mainly homozygous genotype. Leukocytes and platelet count did not differ significantly. Due to the small number of individuals with iron deficiency found among blood donors, the differential diagnosis between the two types of anemia was not possible, even because minor changes were found among hematological parameters with iron deficiency and *α*-thalassemia. Despite this, the study showed the values of hematological parameters, especially MCV and MCH, are lower in donors with iron deficiency, especially when associated with *α*-thalassemia, and therefore, it may be useful to discriminate different types of microcytic anaemia. In conclusion, we believed screening for thalassemia trait should be included as part of a standard blood testing before blood donation. It should be noted that this was the first study to perform the screening for alpha deletions in blood donors from the Manaus region, and further studies are required to look at the effects of donated thalassemic blood.

## 1. Introduction

Thalassemias are the most common monogenic diseases prevalent worldwide. Thalassemias occur in high frequency in Italy, Middle East, India, and South and Southeast Asia. The most common deletional thalassemia alleles are –*α*^3.7^, observed worldwide, and –*α*^4.2^, common in Asian countries [1–3].

Although many regions of the world still have no demonstrated data, current studies show about 7% of the world population have at least one hemoglobin disorder [4, 5].

The current Brazilian population was formed by successive migratory waves from the 16th to 18th centuries (Africans Slaves) and 19th and 20th centuries (Europeans). Many genetic studies over Brazilian population have been documented this heterogeneity with nonuniform European, African, and Amerindian pattern. Northern populations, generally, have higher African and Amerindians contributions when compared to other Brazilian regions [6–11].

Several laboratory tests may be used to detect and diagnose thalassemia, such as hemoglobin electrophoresis, complete blood count, blood smear, iron studies, and DNA analysis (genetic testing) [12].

Alpha-thalassemia carriers are asymptomatic and usually without hematologic abnormalities. Complications are mostly found in beta-thalassemia major and intermediate patients. In silent thalassemic state, carriers are essentially asymptomatic and the complete blood count, hemoglobin electrophoresis, and peripheral smear are usually normal. Slight hypochromia and microcytosis may be evident by microscopic evaluation [13–17].

Likewise, screening for thalassemia may help in providing blood and red blood cell concentrates with maximum functional capacity. In addition, detection of thalassemia in blood donors may reflect the spectrum of the disease in the population.

This is the first report in Manaus and Amazon regions that identifies thalassemia carriers among blood donors at the HEMOAM Foundation.

## 2. Materials and Methods

The studied population sample is composed of 989 first-time blood donors attending at FHEMOAM in Manaus, capital of the Amazonas State, Brazil, between 2016 and 2017. Only donors older than 18 years and, among these, individuals who claimed to be donating blood for the first time were invited to participated in this study.

Peripheral blood samples for the hematological analysis were obtained during a blood donation routine, 4 ml volume in ethylenediaminetetraacetic acid (EDTA) and 4 ml volume in no anticoagulant BD Vacutainer® blood collection tubes. The hematological investigations performed included a full blood count using the automated hematologic analyzer BC-5800 (Mindray, Shenzhe, China); data have been obtained for the overall count of red blood cells (RBCs), concentration of hemoglobin (Hgb), hematocrit (Hct), mean corpuscular volume (MCV), mean corpuscular hemoglobin (MCH), mean corpuscular hemoglobin concentration (MCHC), red cell distribution width (RDW), total leukocytes, and platelet count. Iron and serum ferritin were measured as implemented in the automated A25 platform (BioSystems SA, Barcelona, Spain) using the Ferritin K081 kit (Bioclin) and Iron Serum K017 kit (Bioclin) [18, 19].

Genomic DNA was extracted from the peripheral blood using the HiYield Genomic DNA Extraction kit (BioAmerica Inc., USA). NanoDrop ND-1000 (Isogen Life Science, Netherlands) was used to measure DNA concentration according to the manufacturer's protocol.

All samples were tested to confirm the normal hemoglobin genotype “AA” using TaqMan® SNP Genotyping Assays (Applied Biosystems, Foster City, CA) on a StepOnePlus™ Real-Time PCR System (Applied Biosystems).

PCR was performed as described previously [20] using the QIAGEN Multiplex PCR kit (1000 reactions; Cat no./ID 206143) with minor changes. In brief, PCR was performed using approximately 50 ng of genomic DNA in 50 *μ*L reaction volumes containing 3 mM MgCl_2_; 5x Q-Solution and 0.35 *μ*mol/L of each primer (Table 1), including primers that act as internal controls; 250 *μ*mol/L DNTPs; 2.5 U of Taq polymerase (Qiagen); and RNase-free water (q.s.p). The cycling was performed using *T100® gradient Thermal Cycler* (Bio-Rad, Hercules, California, USA) with an initial 10 minutes hold at 96°C, followed by 34 cycles at 96°C for 45 seconds, 60°C for 45 seconds, and 72°C for 150 seconds and a final extension at 72°C for 10 minutes.

Descriptive statistics were used to hematological data, iron and ferritin status. Differences in continuous variables between two groups were analyzed using the Student *t* test or Mann–Whitney test for Gaussian and non-Gaussian distributed variables, respectively. One-way ANOVA was performed to calculate the average and standard deviation. A *p* value less than or equal to 0.05 was considered statistically significant. All statistical analyses were performed using the GraphPad Prism Software v. 5.0 (GraphPad Prism Software Inc., San Diego, California, USA) and SPSS version 19.

## 3. Results and Discussion

Blood samples from 989 first-time donors, 714 (72.1%) males and 275 (27.9%) females, were analyzed, and 53 of them (5.35%) were positive to –*α*^3.7^ deletion. The –*α*^4.2^ deletion was detected in only one subject. No subjects with –*α*^20.5^, –^SEA^, and –^MED^ deletions were found, suggesting the probable presence of “rare” *α*-thalassemia genotypes in the Amazon region to be as in other Brazilian populations. The results are summarized in Table 2.

The *α*-thalassemia alleles frequency found from the random blood donors' samples is similar to that estimated in other studies from Brazilian population (average among 2.5–8%). Our study showed lower *α*-thalassemia frequency when compared to 14.89% reported in the Uberaba Regional Blood Center's (Hemominas Foundation, Uberaba, Brazil) work, with all cases being heterozygous for the –α^3.7^ deletion [21–23].

This frequency is probably underestimated since the blood donors are generally healthier and therefore have hematological data with values equal to or above normal reference values when compared to attending population at health posts and hospitals, even for normal patients without clinical manifestations.

As expected, the mean of hematological data was significantly lower in *α*-thalassemia carriers (*p* < 0.001). Leukocytes and platelet count did not differ significantly (Table 3). On comparing hematological parameters, independent of gender, there was a most pronounced microcytosis in the cases with homozygous genotype. Microcytosis was found in all homozygous cases, but it is also important to note that normal RBC indices do not rule out *α*-thalassemia carrier [24]. Our results corroborate the national and international reported data, i.e., alpha-thalassemia, mainly the −*α*^3.7^ deletion as one of the main causes of microcytosis and mild or severe hypochromia in individuals without anemia. These results have high impact in the clinic medicine, since these hematological data are often interpreted as indicators of iron deficiency [25, 26].

We propose *α*-thalassemia as a common cause of microcytosis, given the high proportion (42.2%) of the microcytic blood donors carrying −*α*^3.7^ deletions. Randomly, 479 subjects were analyzed to test iron and ferritin. The results showed only 1.04% has iron deficiency (*n* = 5) and very high levels of stored iron (>220 *µ*g/dL) were not observed. Microcytic hypochromic anemia is a common hematological abnormality, and it is usually caused by iron deficiency and/or thalassemia, usually *α* genes deletion. Our results point out to the importance of investigating *α*-thalassemia, besides serum iron levels, to prevent medical intervention and iron therapy unnecessary in subjects with microcytic anemia due to thalassemia [27–29].

Serum iron and ferritin dosages are good indicators of iron storage, enabling the early detection of iron deficiency. Nonetheless, in cases where ferritin levels are within the reference values or increased, other tests such as total iron binding capacity and transferrin saturation may help in better characterization of the type of anemia [30, 31].

In blood banks from Brazil, although there are rigid and regulated techniques, it is still a great problem to enhance the identification of all types and causes of anemia. This is due to the need to use different methodologies in the differential diagnosis [16]. However, several studies have shown good results using hematological parameters of the conventional hemogram [32, 33].

Another interesting finding was the tendency of red cell volume distribution width (RDW) elevation proportionally to the loss of the alpha genes, corroborating with other studies in the literature. The thalassemia patients have smaller and more homogeneous erythrocytes (mainly microcytic), which leads us to correlate high RDW in iron deficiency than in thalassemia [34–36].

We understand the difficulty in screening for alpha-thalassemia genotypes, especially for the final diagnosis, it is necessary to include a technique that is currently expensive and with few people qualified for it. However, we consider that molecular techniques should be included as part of standard blood tests before blood donation. We even think that national policies should be reformulated to select the blood used for healthy transfusion and with no risk.

## Figures and Tables

**Table 1:** Primers used for detection of common deletional determinants of alpha-thalassemia.

Primer code	5′-3′ sequence	Fragment size (base pairs)
LIS1-F	G T C G T C A C T G G C A G C G T A G A T C	∼2503
LIS1-R	G A T T C C A G G T T G T A G A C G G A C T G	
*α*2^3.7^F	C C C C T C G C C A A G T C C A C C C	∼2022
*α*2 R	A G A C C A G G A A G G G C C G G T G	
*α*2^3.7^F	C C C C T C G C C A A G T C C A C C C	∼1800
^3.7/20.5^R	A A A G C A C T C T A G G G T C C A G C G	
SEA-F	C G A T C T G G G C T C T G T G T T C T C	∼1350
SEA-R	A G C C C A C G T T G T G T T C A T G G C	
^4.2^F	G G T T T A C C C A T G T G G T G C C T C	∼1630
^4.2^R	C C C G T T G G A T C T T C T C A T T T C C C	
^20.5^F	G C C C A A C A T C C G G A G T A C A T G	∼1000
^3.7/20.5^R	A A A G C A C T C T A G G G T C C A G C G	
FIL-F	T G C A A A T A T G T T T C T C T C A T T C T G TG	∼1170
FIL-R	A T A A C C T T T A T C T G C C A C A T G T A G C	
MED-F	T A C C C T T T G C A A G C A C A C G T A C	∼807
MED-R	T C A A T C T C C G A C A G C T C C G A C	

**Table 2:** Overall alpha-thalassemia genotypes among the first-time blood donors from FHEMOAM (2016-2017)

Gender	*α*-Globin genotypes					Total
	*αα*/*αα*	−*α*^3.7^/*αα*	*α* ^3.7^/*α*^3.7^	−*α*^4.2^	^−MED, −FIL, SEA, 20.5^	
Male (%)	671 (93.9)	37 (5.2)	05 (0.7)	01 (0.1)	—	714
Female (%)	264 (96.0)	9 (3.3)	02 (0.7)	—	—	275
Total (%)	935 (94.4)	46 (4.7)	07 (0.7)	01 (0.1)	—	989

**Table 3:** Hematologic, serum ferritin, and serum iron characterization between** **−*α*^3.7^ genotypes and gender from the first-time blood donors from FHEMOAM (2016-2017).

Parameters data	*α*-Globin genotypes (all)			*p* value	*α*-Globin genotypes (male)			*p* value	*α*-Globin genotypes (female)			*p* value
	*αα*/*αα*	−*α*^3.7^/*αα*	*α* ^3.7^/*α*^3.7^		*αα*/*αα*	−*α*^3.7^/*αα*	*α* ^3.7^/*α*^3.7^		*αα*/*αα*	−*α*^3.7^/*αα*	*α* ^3.7^/*α*^3.7^	
WBC (×10^3^/*μ*L)	6.92 ± .96	7.21 ± .68	7.16 ± 1.11	0.174	6.99 ± .95	7.33 ± .63	7.19 ± 1.57	0.179	6.68 ± .96	6.84 ± .73	7.11 ± .88	0.802
RBC (×10^6^/mm L)	4.74 ± .43	5.04 ± .35	5.57 ± .76	<0.001	4.87 ± .38	5.11 ± .32	5.76 ± .73	<0.001	4.42 ± .36	4.77 ± .31	5.09 ± .35	<0.001
Hg (g/dL)	14.13 ± 1.28	14.05 ± 1.41	12.11 ± 1.38	<0.001	14.60 ± 1.1	14.47 ± 1.2	12.54 ± 1.4	<0.001	12.99 ± 1.1	12.35 ± .7	11.05 ± .91	0.009
Hct (%)	43.21 ± 3.32	43.13 ± 3.03	42.01 ± 2.66	.591	44.26 ± 2.9	43.81 ± 2.7	42.58 ± 2.5	0.292	40.32 ± 2.7	39.51 ± 2.1	39.1 ± 1.9	0.659
MCV (fL)	90.39 ± 4.42	84.97 ± 4.81	74.49 ± 7.28	<0.001	90.91 ± 4.41	85.83 ± 4.70	74.54 ± 2.89	<0.001	89.12 ± 4.2	81.41 ± 3.6	73.59 ± .5	<0.001
MCH (pg/l)	29.82 ± 1.82	27.87 ± 2.34	21.98 ± 3.09	<0.001	29.99 ± 1.7	28.34 ± 2.2	22.09 ± 3.7	<0.001	29.42 ± 1.9	25.93 ± 1.7	21.69 ± .29	<0.001
MCHC (g/dl)	32.96 ± 1.08	32.77 ± 1.42	29.43 ± 1.82	<0.001	32.99 ± 1.44	33.01 ± 1.43	29.40 ± 2.21	<0.001	32.89 ± 1.2	31.83 ± 1.1	29.48 ± .61	<0.001
RDW (%)	13.27 ± .89	14.59 ± 1.23	16.13 ± 1.51	<0.001	12.43 ± .91	13.85 ± 1.2	16.18 ± 1.4	<0.001	12.88 ± .72	13.52 ± .54	15.11 ± 2.3	<0.001
Platelet (×10^9^/L)	278.3 ± 98.8	261.6 ± 61.8	277.4 ± 92.8	0.461	272.2 ± 87.4	261.3 ± 72.4	257.4 ± 124.5	0.709	293.3 ± 106.6	263.1 ± 54.9	281.7 ± 38.2	0.693
Serum iron (*μ*g/dL)	91.29 ± 19.31	82.15 ± 102.4	60.29 ± 28.2	<0.001	81.9 ± 5.5	76.6 ± 4.5	51.2 ± 27.5	<0.001	120.6 ± 17.5	99.5 ± 30.8	78.5 ± 26.4	<0.001
Serum ferritin (*μ*g/dL)	156.1 ± 47.7	157.4 ± 45.9	118.2 ± 59.66	<0.001	179.1 ± 28.1	169.8 ± 21.4	121.12 ± 79.7	<0.001	88.1 ± 22.6	87.5 ± 26.1	84.4 ± 24.4	.894

## Data Availability

The data used to support the findings of this study may be released upon application to the corresponding author via email (jp-mn@hotmail.com).
